# Increased Th1 and suppressed Th2 serum cytokine levels in subjects with diabetic coronary artery disease

**DOI:** 10.1186/1475-2840-13-1

**Published:** 2014-01-03

**Authors:** Haridoss Madhumitha, Viswanathan Mohan, Mohan Deepa, Subash Babu, Vivekanandhan Aravindhan

**Affiliations:** 1 AU-KBC Research Centre, MIT Campus of Anna University, Chennai 600 044, India; 2 Madras Diabetes Research Foundation & Dr. Mohan’s Diabetes Specialties Centre, WHO Collaborating Centre for Non-Communicable Diseases Prevention and Control, International Diabetes Federation (IDF) Centre for Education, Chennai, India; 3 National Institutes of Health-International Center for Excellence in Research, National Institute for Research in Tuberculosis, Chennai, India

**Keywords:** CAD, T2DM, IL-2, IL-12, IFN-γ, IL-4, IL-5, IL-13

## Abstract

**Background:**

The role played by T helper cytokines under chronic, low grade inflammation as seen in type-2 Diabetes Mellitus (T2DM) and Coronary Artery Disease (CAD) co-morbidity is less well studied. In the present study, we measured the serum levels of both Th1 and Th2 cytokines and correlated it with clinical risk factors for T2DM (Insulin Resistance (IR), Glycated haemoglobin (HbA1c)) and CAD (C-Reactive Protein (CRP), Intima Media Thickness (IMT) and Augmentation index (AGI)) in T2DM subjects with/without CAD.

**Methodology:**

The study subjects were recruited from Chennai Urban Rural Epidemiology Study (CURES). Serum cytokine profile was determined by multiplex cytokine assay in Control (n = 61), T2DM (n = 60), CAD (n = 23) and T2DM-CAD (n = 21) subjects.

**Results:**

T2DM subjects showed a mixed Th1-Th2 profile. CAD subjects presented a Th1 profile with modest Th2 suppression while T2DM-CAD subjects showed enhanced Th1 profile with strong suppression of Th2 cytokines. Both Th1 and Th2 cytokines showed a positive correlation with FPG, HbA1c, hsCRP, IMT and AGI. Logistic regression analysis revealed a significant association of IL-12 (OR = 9.3; 95% CI = 3.2-70.7; p = 0.016), IFN-γ (OR = 2.8; 95% CI = 2.7-2.9, p = 0.010), IL-4 (OR = 2.7; 95% CI 2.7-2.7, p = 0.010), IL-5 (OR = 1.1; 95% CI = 1.0-1.4; p = 0.003) and IL-13 (OR = 2; 95% CI = 1.7-2.6; p = 0.017) with T2DM-CAD.

**Conclusion:**

In conclusion, from the present study it appears that transition from T2DM or CAD to T2DM-CAD co-morbidity is associated with strong down regulation of Th2 cytokines and enhancement of Th1 responses.

## Introduction

Chronic, low grade inflammation with nitrosative stress has now been identified as an essential component of both Type-2 Diabetes Mellitus (T2DM) and Coronary Artery Disease (CAD) [1-3]. The excessive morbidity and mortality seen in T2DM subjects is primarily due to the increased incidence of cardiovascular diseases (including CAD) in these subjects accounting for 22% of total deaths in 2008 and is projected to increase to 26% by 2030 (World Health Statistics, 2008). Apart from the traditional risk factors of T2DM and CAD such as obesity, hypertension and dyslipidemia, chronic inflammation has now emerged as a major risk factor for both these conditions [1]. The presence of activated T-cells in human atherosclerotic plaque (in the case of CAD) and in adipose tissue (in the case of T2DM) has been identified several years ago indicating the involvement of adaptive immunity in these disease conditions [4]. Upon activation, T lymphocytes differentiate into T-helper (Th)1 and Th2 subsets secreting either Th1 (Interferon (IFN)-γ and Interleukin(IL)-2) or Th2 cytokines (IL-4, IL-5 and IL-13) respectively [5]. IL-12 has long been identified as the master controller of Th1 differentiation while recently, IL-33 has emerged as a master regulator of Th2 differentiation [5,6]. The role played by the pro-inflammatory cytokines such as TNF-α, IL-6 and IL-1β in atherogenesis and insulin resistance has been well documented [7-9]. A recent study has indicated IL-6 and activin-A as major risk factors for cardiovascular events and mortality in T2DM subjects [10]. However, less known is the role played by T-cell cytokines under conditions of T2DM and CAD. Even less studied is the role played by these cytokines under conditions of T2DM-CAD co-morbidities. In these subjects, inflammation associated with one condition can augment/synergize with the inflammation associated with the other condition. Previously, we have reported mixed Th1-Th2 serum cytokine profile in subjects with metabolic syndrome (MS), a major risk factor for T2DM (if not present already) and CAD [7]. In the present study, we measured the serum levels of both Th1 and Th2 cytokines and correlated it with clinical risk factors for T2DM (Insulin Resistance (IR), Glycated haemoglobin (HbA1c)) and CAD (C-Reactive Protein (CRP), Intima Media Thickness (IMT) and Augmentation index (AGI)) in subjects with T2DM with/without CAD.

## Methodology

The study subjects were recruited from the Chennai Urban Rural Epidemiological Study (CURES), an ongoing epidemiological study conducted on a representative population (≥20 years old) of Chennai (formerly Madras), the fourth largest city in India. The methodology of the study has been published elsewhere [11]. In brief, 26,001 individuals were recruited for the Phase 1 of the urban component of CURES, using a systematic random-sampling technique. Fasting capillary blood glucose was determined using an OneTouch_ Basic_ glucometer (Lifescan, a Johnson & Johnson Company, Milpitas, CA) in all subjects. In Phase 2 of CURES, all the known diabetes subjects in Phase 1 were invited to the centre for detailed studies on vascular complications. In Phase 3 every 10th subject in Phase 1 was invited for clinical, biochemical, microvascular, and macrovascular examinations. For the present study, the following subjects were randomly selected from Phase 3 of CURES and were allocated into the following groups:

Group 1 (n = 61): Subjects who had normal glucose tolerance (Control)

Group 2 (n = 60): Subjects with known T2DM

Group 3 (n = 23): Subjects with known CAD

Group 4 (n = 21): Subjects with both T2DM and CAD

### Inclusion and exclusion criteria

The inclusion criteria were patients within the normal range of white blood cells to minimize the confounding effect of infections. The exclusion criteria were patients with type-1 diabetes and patients with a previous diagnosis of urolithiasis, liver cirrhosis, congestive heart failure, chronic lung diseases, chronic infections or viral hepatitis. Institutional ethical committee approval from the Madras Diabetes Research Foundation Ethics Committee was obtained (Ref No-MDRF-EC/SOC/2009//05) and written informed consent was obtained from all the study participants. Subjects with self reported diabetes receiving treatment were classified as “known diabetes subjects.” CAD was diagnosed based on positive medical history (documented myocardial infarction (MI), angina pectoris and coronary artery bypass graft) and/or ischemic changes on a conventional 12-lead ECG which included ST-segment depression (Minnesota codes 1-1-1 to 1-1-7) or Q-wave changes (Minnesota codes 4–1 to 4–2) [12]. Absence of CAD was based on absence of history of angina or MI and normal ECG.

### Anthropometric measurements and biochemical parameters

Anthropometric measurements including height, weight, and waist circumference, were obtained using standardized techniques. The body mass index (BMI) was calculated as the weight in kilograms divided by the square of height in meters. Fasting plasma glucose (FPG) (glucose oxidase-peroxidase method), serum cholesterol (cholesterol oxidase-peroxidase- amidopyrine method), serum triglycerides (glycerol phosphate oxidase-peroxidase-amidopyrine method), high density lipoprotein cholesterol (HDL-C) (direct method-polyethylene glycol-pretreated enzymes), and creatinine (Jaffe’s method) were measured using a Hitachi-912 Autoanalyser (Hitachi, Mannheim, Germany). The intra- and inter assay coefficient of variation for the biochemical assays ranged between 3.1% and 5.6%. Glycated hemoglobin (HbA1c) was estimated by high pressure liquid chromatography using a variant machine (Bio-Rad, Hercules, CA). The intra- and inter-assay coefficient of variation of HbA1c was less than 5%. The plasma concentrations of high-sensitivity C-reactive protein (hsCRP) were measured by high sensitive nephelometric assay. The intra- and the inter-assay coefficients of variation for hsCRP were 4.2% and 6.8%, respectively, and the detection limit was 0.15 mg/L. Both the intra and inter assay variations were determined in our biochemical lab.

### Measurement of intima media thickness and arterial stiffness

The method used for measurement of carotid intima media thickness (IMT) has been previously described [13]. The intima plus medial thickness of the right common carotid artery was determined using a high-resolution B-mode ultrasonographic system (Logic 400 GE, Milwaukee, Wisconsin) with an electrical linear transducer mid frequency of 7.5 MHz. The axial resolution of the system was 0.3 mm. The images were recorded, as well as photographed. Scanning was performed for an average of 20 minutes. IMT as defined by Pignoli and Longo [13] was measured as the distance from the leading edge of the first echogenic line to the second echogenic line. The mean of the 6 IMT measurements (3 from the far wall and 3 from the near wall) was used as the representative value for each subject. The scanning was done using fine manipulations of the transducer, to visualize, with maximum clarity, the double-line pattern of the IMT both at the near and far wall of the artery.

Arterial stiffness was measured using the Sphygmocor apparatus (Sphygmocor BPAS-1; PWV Medical, Sydney, Australia). In brief, a high-fidelity micromanometer (SPC-301; Millar Instruments, Houston, Texas) was used to flatten but not occlude the right radial artery, using gentle pressure. When the 2 surfaces are flattened, circumferential pressures are equalized and an accurate pressure waveform can be recorded. Data were collected directly into a portable microcomputer. The system software allowed on-line recording of the peripheral waveform, which was assessed visually to ensure that the best possible recording was obtained and that artifacts from movement were minimized. After 20 sequential waveforms had been acquired, the integral software was used to generate an averaged peripheral and corresponding central waveform that was used for the determination of the Agumentation Index (AGI). AGI was defined as the difference between the first and second peaks of the central arterial waveform, expressed as a percentage of the pulse pressure [14].

### Estimation of levels of serum cytokines

The levels of cytokines (IL-2, IL-12, IFN-γ, IL-4, IL-5, and IL-13) in the serum were measured using a Bio-Plex multiplex cytokine bead assay system (Bio-Rad). The lower detection limits were 16 pg/mL for IL-2, 0.3 pg/mL for IL-4, 2.08 pg/mL for IL-5, 2.78 pg/mL for IL-12, 2.22 pg/mL for IL-13, 2.14 pg/mL for IFN- γ. The intra- and interassay coefficients of variation for multiplex assay were less than 5%.

### Statistical analysis

Data are expressed as geometric mean values. Student t-test was used to compare groups for continuous variables, whereas χ2 test or Fisher exact test (as appropriate) was used to compare proportions. The Mann–Whitney U test was used in case of non-normally distributed parameters to compare means. Spearman correlation analysis was carried out to determine the association of serum cytokine levels with clinical parameters. Multivariate Logistic regression analysis was used to determine the association of serum cytokines with T2DM-CAD. Kruskal-Wallis test was used for multiple parameters that did not show normal distribution. Multiple comparisons were corrected using the Holm’s correction for each set of analysis. All the analyses were done using SPSS statistical package (Version 20.0; SPSS, Chicago, IL) and P value less than 0.05 was considered significant.

## Results

Table 1 shows the clinical and biochemical characteristics of the study subjects. T2DM subjects were significantly older and had higher BMI compared to controls (p < 0.05). As expected they had significantly higher fasting plasma glucose and HbA1C (p < 0.0001). Dyslipidemia and hypertension was observed in 42% and 32% of the subjects respectively. Their renal parameters such as urea, creatinine and microalbuminuria were within the normal limits (Urea- 10–20 mg/dL; Creatinine- 0.7-1.3 mg/dL; Microalbuminuria: <30 mg). The CAD subjects were relatively younger and had normal BMI compared to T2DM subjects. This observation is in line with the previous studies which have reported premature CAD in south Indian population and the higher susceptibility of this ethnic group for CAD [15]. These subjects were non-diabetic and hence had normal fasting plasma glucose and HbA1c levels. Dyslipidemia and hypertension was observed in 26% and 13% respectively. Urea, creatinine and microalbuminuria levels were within the normal range. The subjects with T2DM-CAD co-morbidities were significantly older compared to the control and CAD subjects. 24% of them were obese, 67% had hypertension and 38% showed dyslipidemia. They had significantly higher fasting plasma glucose and HbA1c values. However the renal parameters were within the normal limits.

**Table 1:** Clinical and biochemical characteristics of the study subjects

**Clinical parameters**	**Control (NGT)**	**T2DM**	**CAD**	**T2DM_CAD**
	**n = 61**	**n = 60**	**n = 23**	**n = 21**
Age (Years)	39.6 ± 13.7	56.4 ± 13.9^a***^	38.7 ± 15.8	60.9 ± 10.7^c***^
Body mass index (kg/m^2^)	23.19 ± 4.8	25.5 ± 5.2	21.1 ± 4.3	23.2 ± 4
Systolic BP (mm Hg)	114 ± 9.5	125 ± 2.2^a**^	122.5 ± 20.6	142.4 ± 19.9^b**c**^
Diastolic BP (mm Hg)	74.98 ± 8.5	77.2 ± 9.0	71.8 ± 10.03	87.3 ± 11.04^b*^
Fasting plasma glucose (mg/dL)	84.9 ± 9.1	158.9 ± 67.5^a***^	85.8 ± 12.5	136.2 ± 41.4^c***^
Glycated haemoglobin (%)	5.3 ± 0.37	8.5 ± 1.9^a***^	5.4 ± 0.5	7.6 ± 0.8^c**^
Total serum cholesterol (mg/dL)	174.5 ± 34.08	173.3 ± 46.02	188.2 ± 81.2	182.3 ± 37.1
Serum triglycerides (mg/dL)	108.2 ± 51.1	168.4 ± 100.4^a***^	105.1 ± 54.6	140.1 ± 77.4
HDL-cholesterol (mg/dL)	43 ± 7.9	40 ± 9.1	47.7 ± 12.6	41.9 ± 11.9
LDL-cholesterol (mg/dL)	107.7 ± 28.7	98.5 ± 37.9	119.5 ± 73.4	112.4 ± 33.3
VLDL-cholesterol (mg/dL)	20.2 ± 9.4	49.6 ± 51.5^a***^	20.9 ± 10.8	23.5 ± 11.4
Urea	20.5 ± 5.2	23.5 ± 8.7	20.8 ± 5.7	22.3 ± 4.1
Creatinine	0.85 ± 0.16	4.6 ± 11.7	0.8 ± 0.16	0.8 ± 0.24
Microalbuminuria (mg/dL)	16.4 ± 48.4	22.1 ± 33.9	15.1 ± 20.2	15 ± 16.4

For all the parameters mean ± SD is reported. ^a^Comparison between Control and T2DM, ^b^Comparison between T2DM and T2DM-CAD, ^c^Comparison between CAD and T2DM-CAD. *P < 0.05, **P < 0.01, ***P < 0.0001.

**Figure 1:**
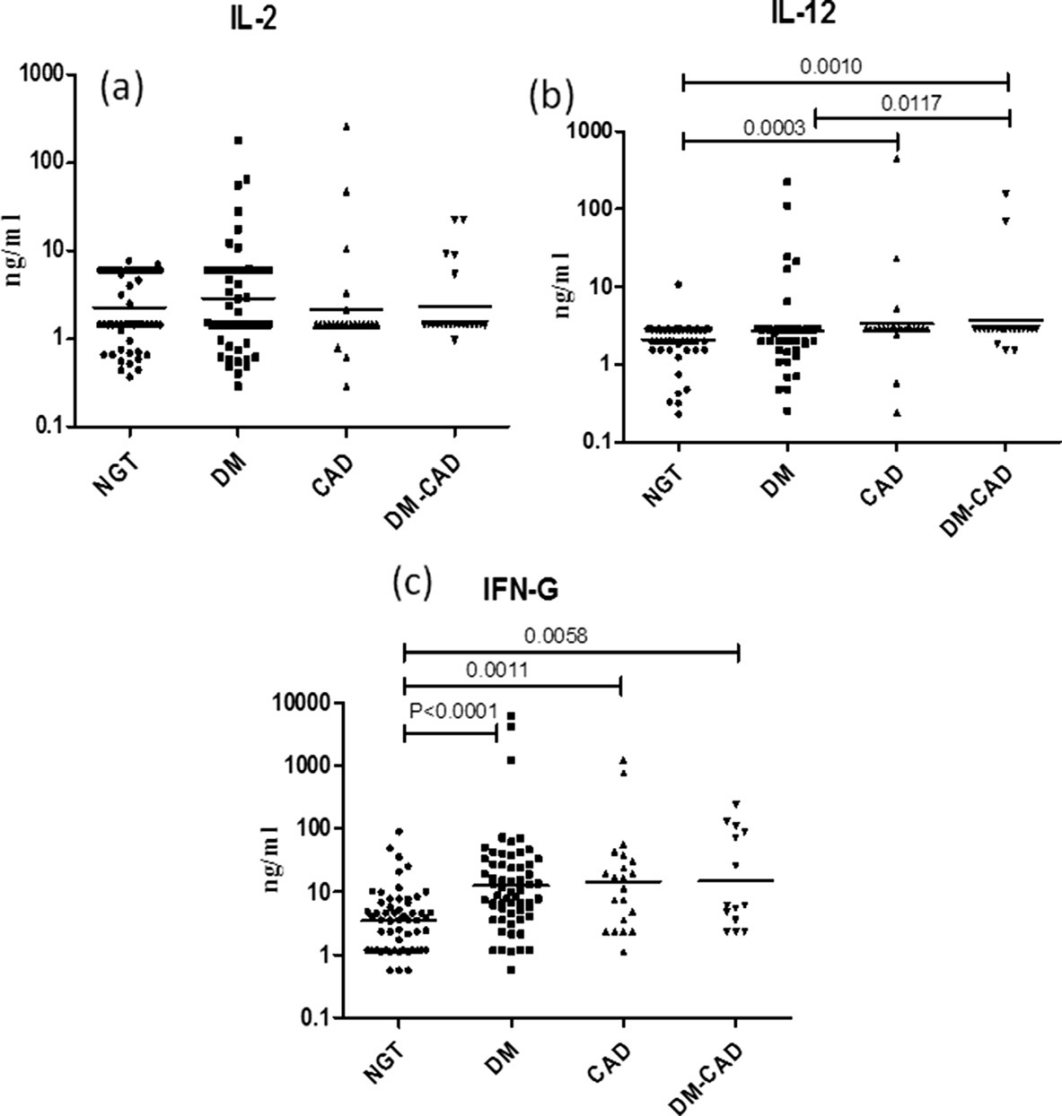
**Serum levels of Th1 cytokines (IL-2, 12 and IFN-γ) in the study groups.** Serum levels of IL-2 **(a)**, IL-12 **(b)** and IFN-γ **(c)** were determined in control (NGT), T2DM, CAD and T2DM-CAD subjects by multiplex cytokines assay. Each dot represents individual values with the horizontal line representing the geo mean. Statistical significance was determined by non-parametric Kruskal–Wallis one-way analysis of variance and p < 0.05 was considered significant.

**Figure 2:**
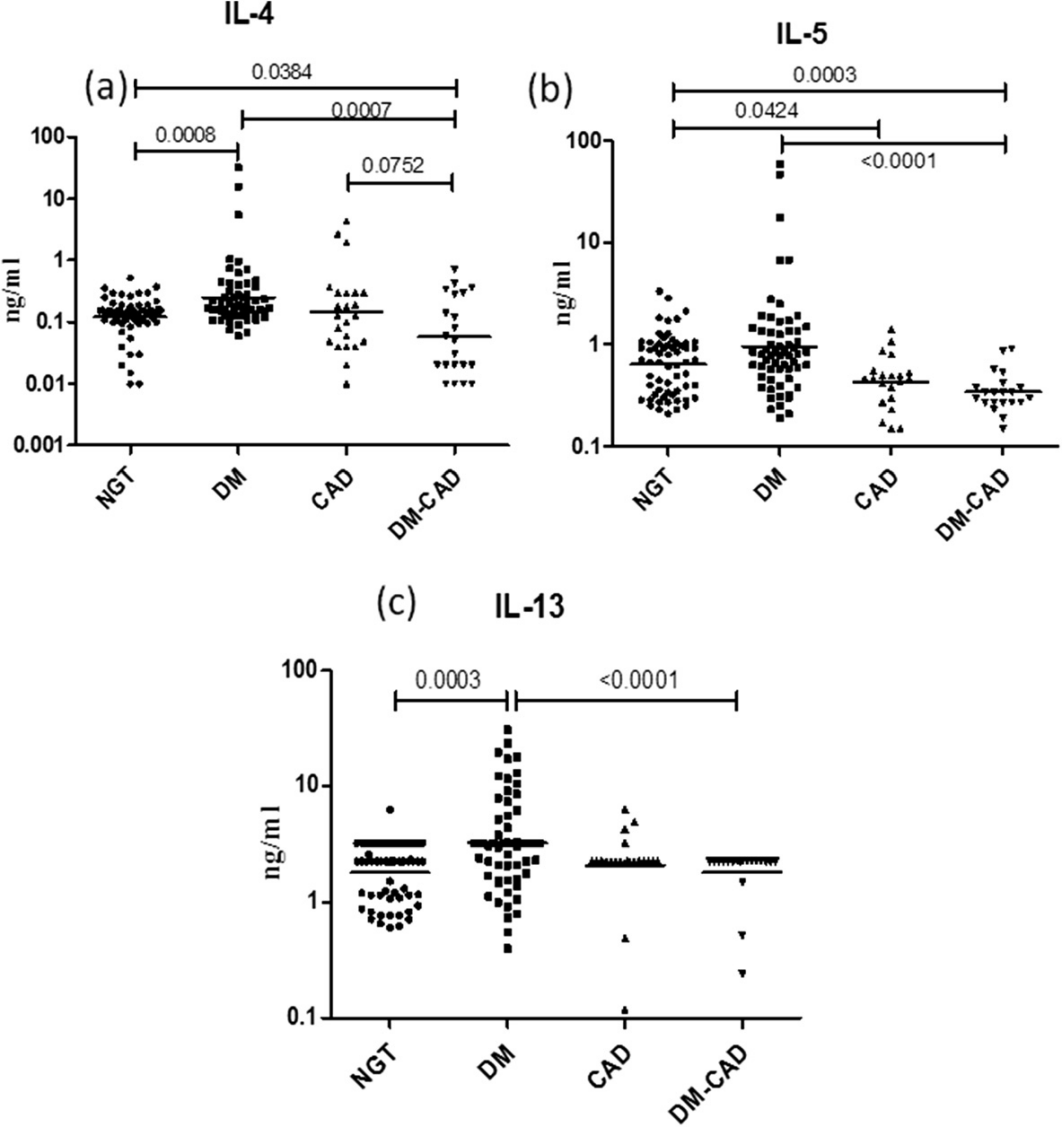
**Serum levels of Th2 cytokines (IL-4, 5 and 13) in the study groups.** Serum levels of IL-4 **(a)**, IL-5 **(b)** and IL-13 **(c)** were determined in control (NGT), T2DM, CAD and T2DM-CAD subjects by multiplex cytokines assay. Each dot represents individual values with the horizontal line representing the geo mean. Statistical significance was determined by non-parametric Kruskal–Wallis one-way analysis of variance and p < 0.05 was considered significant.

To determine serum Th1 cytokine profile the levels of IL-2, IL-12 and IFN-γ were estimated in the study groups (Figure 1). As can be seen in Figure 1, significantly high levels of IFN-γ (but not IL-12) were seen in the T2DM group while significantly elevated levels of both IL-12 and IFN-γ were seen in CAD and T2DM-CAD groups compared to controls. Overall the levels of IFN-γ were not significantly different between T2DM, CAD and T2DM-CAD groups. IL-2 levels were not significantly different between the groups (Fig 1a). Next, the levels of Th2 cytokines IL-4, IL-5 and IL-13 were determined in the study groups (Figure 2). As can be seen in Figure 2, significantly elevated levels of both IL-4 and IL-13 were seen in the T2DM group while significantly low levels of IL-5 were seen in the CAD group, compared to controls. T2DM-CAD subjects had significantly lower levels of all the three Th2 cytokines compared to that of T2DM group, indicating strong Th2 suppression.

Additional file 1: Table S1 shows Spearman’s correlation between the mean cytokine levels and the T2DM and CAD risk factors. IL-2 showed a negative correlation with Diastolic BP (r = −0.158; P = 0.046) and positive correlation with AGI (r = 0.577, P < 0.0001). IL-12 showed a negative correlation with HbA1C (r = −0.212, P = 0.011) and VLDL (r = −0.183, P = 0.026). IFN-γ showed a positive correlation with FPG (r = 0.228; P = 0.004) and HbA1C(r = 0.339; p < 0.0001) whereas negative correlation with IMT (r = −0.328, p = 0.011). IL-4 showed a positive correlation with Age (r = 0.308, P < 0.0001), FPG (r = 0.314; P < 0.0001), HbA1C (r = 0.439; P < 0.0001), VLDL (r = 0.234, P = 0.004), AGI (r = 0.313, P = 0.047) and negative correlation with CHO (r = −0.231; P = 0.003) and LDL (r = −0.337; p = <0.0001). IL-5 showed a positive correlation with FPG (r = 0.184; P = 0.02) and HbA1C (r = 0.243; p = 0.004) whereas a negative correlation with IMT(r = −0.376, p = 0.003) and AGI (r = −0.366, P = 0.018). IL-13 showed a positive correlation with Age (r = 0.278, P = 0.001), FPG (r = 0.244; P = 0.002), HbA1C (r = 0.312; p < 0.0001), Triglycerides (r = 0.161; P = 0.041), VLDL (r = 0.201; P = 0.014) and IMT (r = 0.341, p = 0.008).

Additional file 2: Table S2 shows the results of multivariate logistic regression analysis. With NGT and T2DM as dependent variables and the cytokines as independent variables IL-13 (OR = 4.8, 95% CI 2.7-11.7, p = 0.048) showed a strong and IL-12 showed a weak (OR = 1.9, 95% CI 1.5-2.7, p = 0.043) association with T2DM. With NGT and CAD as dependent variable, IL-2 (OR = 1.8, 95% CI = 1.5-2.4, p = 0.010), IFN-γ (OR = 2.9, 95% CI = 2.7-3.2, p = 0.042) and IL-4 (OR = 2.7, 95% CI = 2.7-2.8, p = 0.029) showed a significant association with CAD. With CAD and T2DM-CAD as dependent variable only IL-4 (OR = 2.7, 95% CI = 2.6-2.7, p = 0.036) showed an association with T2DM-CAD. However using T2DM and T2DM-CAD as dependent variables, IL-12 (OR = 9.3;95% CI = 3.2-70.7;p = 0.016), IFN-γ (OR = 2.8; 95% CI = 2.7-2.9, p = 0.010), IL-4 (OR = 2.7; 95% CI 2.7-2.7, p = 0.010), IL-5 (OR = 1.1;95% CI = 1.0-1.4; p = 0.003) and IL-13 (OR = 2;95% CI = 1.7-2.6; p = 0.017) showed significant association with T2DM-CAD.

## Discussion

Both T2DM and CAD are characterized by chronic, low grade non-specific inflammation, which has long been associated with sterile innate immune responses mediated by heightened levels of serum pro-inflammatory cytokines (TNF-a, IL-6 and IL-1b) [16]. However, recent identification of T cells in the primary organs of inflammation namely adipose (in the case of T2DM) and atherosclerotic plaque (in the case of CAD) had induced renewed interest in studying T cell cytokines in these disease conditions. Recent studies have identified the potential of using immunomodulatory therapeutics to treat cardiovascular events among T2DM subjects which included statins, monoclonal antibodies and anti-inflammatory agents [17]. Towards this end, we studied both Th1 and Th2 cytokines in subjects with T2DM, CAD and T2DM-CAD co-morbidities. The major findings are as follows: 1.T2DM subjects showed a mixed Th1-Th2 profile, 2. CAD subjects presented a Th1 profile with reduced levels of IL-5, 3. T2DM-CAD subjects showed Th1 profile with strong suppression of all the Th2 cytokines 4. Both Th1 and Th2 showed a positive correlation with FPG, HbA1c, hsCRP, IMT and AGI and 5. Logistic regression analysis showed a significant association of IL-12, IFN-γ, IL-4, IL-5 and IL-13 with T2DM-CAD even after adjusting for age and gender.

In our previous study, we have reported a mixed Th1/Th2 profile in subjects with Metabolic Syndrome (MS) [7]. In the present study, we observed a similar “mixed Th1/Th2 profile” in T2DM subjects. This similarity in the serum cytokine profile between these related conditions could be due to the common risk factors associated with them (central obesity, dyslipidemia and IR). In general, the association of Th1 cytokines with adipose inflammation is well documented in both animals and humans [18,19]. Obese IFN-γ deficient animals had shown significant reduction in the expression of mRNA-encoding inflammatory genes in adipose tissue (AT) indicating the role of IFN- γ in adipose inflammation and IR [18]. Pacifico et al., has demonstrated an increase in IFN-γ secreting CD4^+^ T cells in obese children [19]. Furthermore, these CD4^+^ T cells showed a positive association with leptin, insulin and HOMA-IR [19]. Apart from IFN- γ, elevated levels of IL-12 have been observed in patients with T2DM and were found to be strongly associated with IR [20]. These reports signify the role of Th1 cytokines in AT inflammation and IR. However, the exact mechanism by which Th1 cytokines bring about IR is not clearly known. It could be due to their interference with the insulin signalling and insulin stimulated glucose uptake eventually leading to IR. Along with Th1 cytokines, a significant up-regulation of Th2 cytokines (IL-4, IL-5, and IL-13) was also seen in T2DM subjects. Data available on the Th2 serum cytokine profile in T2DM subjects is scant. In a recent study, decreased serum levels of IL-13 in T2DM subjects was reported which was implicated in impaired glucose uptake and metabolism [21]. Chang et al., have demonstrated the role of IL-4 in improving insulin sensitivity and glucose tolerance in an animal model of diet induced obesity [22]. Thus with the current finding and in the light of the available literature the increased levels of Th2 cytokines in T2DM implicates a counter measure to inhibit Th1 immunity and there by IR.

Next, we evaluated the T cell cytokine profile in CAD. Apart from its role in IR, T-cells also play a critical role in the initiation, progression and rupture of atherosclerotic plaque leading to CAD and other cardiovascular complications. The presence of T-cells in atherosclerotic plaques has been identified long back in the 1980s [4]. Hansson and co-workers have reported that most of the atherosclerotic plaque cells express HLA-DR, indicating continuous activation by IFN-γ [23]. The same group has demonstrated the expression of IL-2 and IFN-γ in a large proportion of the plaque [24]. In ApoE knock-out mice, IFN-γ was shown to potentiate atherosclerosis through both local and systemic effects [25]. IFN-γ has also been proposed as a component of five panel marker for the prediction of CAD in symptomatic patients referred for coronary angiography [26]. In contrast to Th1 cells, Th2 cells are rarely detected within the atherosclerotic lesions [27]. In line with these reports, we found enhanced Th1 cytokine profile in CAD subjects with significant decrease in IL-5 levels. Thus accumulating evidence suggests that an imbalance in the Th1/Th2 cytokines with enhanced Th1 immune response and suppressed Th2 response, has an important role in the pathogenesis of CAD [28].

Even though the pathophysiology of insulin resistance and atherosclerosis may have a common inflammatory basis, the nature/type of inflammation per se might be different between these conditions. In the present study, T2DM-CAD subjects showed an enhanced Th1 polarization similar to that of CAD subjects with further reduction in their Th2 cytokine levels. In a previous study, it has been reported that Th1 to Th2 ratio shifted more towards Th1 dominance in both acute coronary syndrome and stable CAD [29]. The exact cause for low levels of Th2 cytokines in T2DM-CAD is currently not known. Recently, autoantibodies have been implicated in the lower levels of serum IL-5 as seen in CAD [30]. It would be interesting to see whether autoantibodies against IL-4 and IL-13 could account for the low levels of these cytokines as seen in T2DM-CAD subjects. Till now to the best of our knowledge even animal studies looking at Th1-Th2 responses under the co-morbidity of T2DM and CAD are not available to draw a corollary for our findings.

## Conclusion

In conclusion, from the present study and other available data, it appears that transition from T2DM/CAD to T2DM-CAD co-morbidity is associated with strong down regulation of Th2 cytokines and enhancement of Th1 responses. The major limitation of our study is the limited sample size of the CAD and T2DM-CAD group and its cross-sectional nature, which means that no cause and effect relationship can be drawn. Nevertheless, this study gains importance in the context of it being conducted in a high-risk ethnic population where no reports on this topic are currently available.

## Abbreviations

AGI: Augmentation index; AT: Adipose tissue; BMI: Body mass index; FPG: Fasting plasma glucose; HDL: High density lipoprotein; CAD: Coronary artery disease; CURES: Chennai urban rural epidemiological study; HbA1C: Glycated haemoglobin; hsCRP: High sensitivity C: reactive protein; IFN-γ: Interferon-gamma; IL: Interleukin; IMT: Intima media thickness; IR: Insulin resistance; LDL: Low density lipoprotein; MS: Metabolic syndrome; NGT: Normal glucose tolerance; OR: Odds ratio; T2DM: Type 2 diabetes mellitus; Th1: T helper 1; Th2: T helper 2; VLDL: Very low density lipoprotein

## Competing interests

The authors declare that they have no competing interests.

## Authors’ contributions

Conceived and designed the experiments: VA VM MD. Provided reagents and technical help: SB. Performed the experiments: HM. Analyzed the data: VA and HM. Wrote the paper: HM. All authors read and approved the final manuscript.

## Authors’ information

HM is a PhD scholar and VA is a Scientist at AU-KBC Research Centre, MIT campus of Anna University, Chennai, India. VM is the Chairman and Chief Diabetologist of Dr. Mohan’s Diabetes Specialities Centre and Director of Madras Diabetes Research Foundation. MD is an epidemiologist in Madras Diabetes Research Foundation, Chennai, India. SB is a Scientist in National Institutes of Health-International Centre for Excellence in Research, National Institute for Research in Tuberculosis, Chennai, India.

## Supplementary Material

Additional file 1: Table S1 Spearman’s Correlation analysis of TH1/TH2 cytokines with the T2DM and CAD risk factors. Description of data: This table contains the results (r, p value) of spearman’s correlation analysis of TH1/TH2 cytokines with the T2DM and CAD risk factors. Click here for file

Additional file 2: Table S2 Logistic Regression Analysis with disease phenotype as dependent variable. Description of data: This table contains the results (Odds Ratio (OR), p value, 95% confidence interval (C.I)) of Logistic Regression Analysis performed with disease phenotype as dependent variable. Click here for file
